# Biological Fluid Microsampling for Therapeutic Drug Monitoring: A Narrative Review

**DOI:** 10.3390/biomedicines11071962

**Published:** 2023-07-12

**Authors:** Alessia Cafaro, Matteo Conti, Federica Pigliasco, Sebastiano Barco, Roberto Bandettini, Giuliana Cangemi

**Affiliations:** 1 Chromatography and Mass Spectrometry Section, Central Laboratory of Analysis, IRCCS Istituto Giannina Gaslini, 16147 Genoa, Italy; alessiacafaro@gaslini.org (A.C.); federicapigliasco@gaslini.org (F.P.); robertobandettini@gaslini.org (R.B.); giulianacangemi@gaslini.org (G.C.); 2 Public Health Department, Imola Local Unit, Regione Emilia-Romagna Healthcare Service, 40026 Imola, Italy; matteo.conti@ausl.imola.bo.it

**Keywords:** microsampling, drug monitoring, narrative review, liquid chromatography tandem mass spectrometry

## Abstract

Therapeutic drug monitoring (TDM) is a specialized area of laboratory medicine which involves the measurement of drug concentrations in biological fluids with the aim of optimizing efficacy and reducing side effects, possibly modifying the drug dose to keep the plasma concentration within the therapeutic range. Plasma and/or whole blood, usually obtained by venipuncture, are the “gold standard” matrices for TDM. Microsampling, commonly used for newborn screening, could also be a convenient alternative to traditional sampling techniques for pharmacokinetics (PK) studies and TDM, helping to overcome practical problems and offering less invasive options to patients. Although technical limitations have hampered the use of microsampling in these fields, innovative techniques such as 3-D dried blood spheroids, volumetric absorptive microsampling (VAMS), dried plasma spots (DPS), and various microfluidic devices (MDS) can now offer reliable alternatives to traditional samples. The application of microsampling in routine clinical pharmacology is also hampered by the need for instrumentation capable of quantifying analytes in small volumes with sufficient sensitivity. The combination of microsampling with high-sensitivity analytical techniques, such as liquid chromatography coupled with tandem mass spectrometry (LC-MS/MS), is particularly effective in ensuring high accuracy and sensitivity from very small sample volumes. This manuscript provides a critical review of the currently available microsampling devices for both whole blood and other biological fluids, such as plasma, urine, breast milk, and saliva. The purpose is to provide useful information in the scientific community to laboratory personnel, clinicians, and researchers interested in implementing the use of microsampling in their routine clinical practice.

## 1. Introduction

Microsampling has emerged as a promising tool for collecting biological fluid samples and has proven to be a suitable strategy for the therapeutic drug monitoring (TDM) of many drugs [1,2]. TDM is a specialized area of laboratory medicine that concerns the personalization of therapies. In particular, TDM refers to the measurement of drugs concentrations in biological liquids to optimize their efficacy, possibly modifying the dose of the drug to keep the plasma concentration within a therapeutic range. This is to reduce the risk of unwanted or toxic effects and increase the benefits of the drug for a specific patient [3]. TDM is especially important in special populations, such as pediatric patients, elderly patients, and patients on polypharmacy, because the pharmacokinetic (PK) profile of drugs can be altered by many physiological and pathological factors [3,4]. TDM is already successfully applied in clinical routines for several classes of drugs. For some drugs, TDM is not yet an established practice, but the availability of methods for monitoring drug levels could certainly be a very useful tool for studying possible pharmacokinetic and/or pharmacodynamic differences in special populations.

Conventional venipuncture is currently the sampling used in clinical practice for TDM. Typically, large volumes of biological fluid samples (>1 mL) are collected, requiring multi-step preparation to obtain the cleanest samples for analysis [2]. The collection of large volumes of blood is not suitable for some clinical settings, such as for pediatric and, in particular, neonatal patients [5]. Microsampling offers several practical advantages over traditional samples, such as minimal invasiveness for patients and simplified logistical requirements. Although equivalence with or without correction factors has been demonstrated in many cases [5,6,7,8,9], it is necessary to validate drug-specific reference/target ranges for each microsampling device. In fact, because of possible differences in the drug concentrations among alternative matrices (capillary blood, urine, breast milk, saliva) [10] and possible interactions of analytes with filtration or adsorption materials, which must be evaluated during method development [11], the reference/target ranges established for TDM in plasma cannot be transferred directly to microsamples [12]. Plasma and/or whole blood are the “gold standard” matrices for TDM, depending on the distribution characteristics of the drugs, but in some cases, alternative matrices also can be applied to TDM.

Dried blood spots (DBS), commonly used worldwide for newborn screening, are obtained by pricking the heel or finger with a lancet and represent a safer and more comfortable procedure than conventional venipuncture [13]. DBS can also be collected independently, as in the case of blood glucose self-testing in diabetic patients, and unlike conventional samples, it can be easily stored and shipped without the need for dry ice [14]. Dried micro samples obtained from biological fluids other than blood, such as dried plasma spots (DPS) [15], dried urine spots (DUS) [16], dried breast milk spots (DBMS) [17], and dried saliva spots (DSS) [18], can be useful for pharmacokinetic studies by overcoming the general requirements of wet samples, such as the need for centrifugation, separation, aliquots, and storage under freezing conditions. 

Currently, the application of microsampling in routine clinical pharmacology is still limited [19,20], mainly because of the need for instrumentation capable of quantifying analytes in very small volumes with sufficient sensitivity, such as liquid chromatography coupled with tandem mass spectrometry (LC-MS/MS), which are found only in specialized centers. LC-MS/MS instrumentation is sensitive enough to enable microsample drug assays and has already enabled their application in routine practice [21], for example, for immunosuppressants [6,20,22,23,24]. Automated dried spot processing devices directly coupled to an LC-MS system with integrated direct elution and extraction steps [25,26] have been successfully applied to the analysis of many drugs, such as antiretrovirals [27], antimycotics [28,29], and antiepileptics [30]. It has been demonstrated that paper spray mass spectrometry (PS-MS) [31] allows for the direct determination of drugs, such as immunosuppressants at part per billion (ppb) levels, from dried spots [22,32].

The use of microsampling, which usually allows low-cost shipping, can facilitate access to this type of analysis even for small centers that cannot afford the significant costs of these instruments.

This manuscript offers a concise review of currently available microsampling techniques and highlights the critical issues that can be commonly encountered when using microsamples from different matrices in pharmacokinetics and TDM.

## 2. Blood Microsamples

DBS have been used in newborn screening since the sixties [33] and in many other bioanalytical fields, such as elemental analysis [16,34], nucleic acids research [35], forensic toxicology [36], proteomics, genomics, and metabolomics [37]. DBS have also been used for the TDM of several classes of drugs, such as antiepileptics drugs (AEDs) [38,39,40], antiretrovirals [41], anticancer drugs [42,43,44], immunosuppressants [11,45], antibiotics [9], antituberculosis [46], and neuroactive drugs [47,48,49,50]. Using DBS is a very convenient option compared with traditional sampling, but drug concentrations in capillary blood may be slightly different from those measured in venous blood [51]; therefore, drug-specific reference intervals are needed to implement DPS-based TDM in clinical practice [12]. Generally, in the dry matrix, given the absence of water, drugs are more stable, but the stability in DBS must be verified for each specific analyte. For example, the stability of ceftolozane is more limited in DBS than in liquid blood [9]. Different types of filter paper can show different matrix effects on the analytes [52]. Interestingly, different matrix effects in the quantification of antipsychotic drugs and their metabolites were observed when results obtained with various cellulose-based untreated filter papers—such as the Whatman^®^ 903 Protein Saver Card or the Fast Transient Analysis (FTA^®^) Drug Metabolism and Pharmacokinetic (DMPK) type C Card—were compared [53]. 

The main issue for drug quantification in DBS is the hematocrit (Hct) effect. Hct is the percentage of red blood cell volume in blood and strongly influences blood viscosity, blood droplet volume, blood droplet migration on the paper substrate, drying time, homogeneity, and spot size, affecting the accuracy and precision of drug quantification. Different Hct values may compromise the reproducibility of the analysis because of the uneven migration of cells and fluids on the paper structure [54,55]. Many attempts have been made to solve the Hct issue [56]. Abu-Rabie et al. (2015) showed that the overall bias given by Hct includes an Hct-based area bias, an Hct-based recovery bias, and an Hct-based matrix effect bias [57]. One example is whole spot analysis, which could overcome the problem of uneven blood distribution on the paper substrate, but the exact volume of blood drawn must be known [58].

The quantification of drugs in microsamples can also be strongly influenced by pre-analytical variables, such as the type of solvent used for drug extraction [42,44,45,48,59] or the way the internal standard (IS) is added to the samples [57,60]. Despite some novel techniques to address the IS having been proposed in the literature, such as the TouchSpray^®^ [60] or the post-column infusion modality (PCI-IS) [61,62], the IS is usually pre-diluted into the extraction solvents. Placing the IS on the DBS and drying it before extraction should be considered the most reliable method to verify drug recovery. Sonication, heating, and the addition of water before extraction for partial rehydration of samples can greatly improve extraction efficiency. Interestingly, it has been observed in several cases that paper substrates can retain proteins, lipids, and phospholipids, providing very clean extracts from dried microsamples, thus improving the performance of LC-MS/MS in analyzing the corresponding liquid microsamples [63].

Several methods for TDM of different classes of drugs are reported in the literature. For example, TDM is used for different AEDs to optimize dosing in individual patients. DBS dosing appears to be a viable alternative to conventional TDM on plasma. Pohanka et al. (2014) [40] developed and validated an LC-MS method for the measurement of valproic acid in dried blood spots. The use of blood samples ranging in size from 20 to 100 μL did not yield significantly different valproic acid concentrations, and the method proved robust in the 30–60% hematocrit range. A comparison between DBS and plasma was performed, and plasma concentrations were significantly higher than DBS, emphasizing the need to create method-specific reference ranges for each analysis. LC-MS/MS methods for the quantitation in DBS were developed also for topiramate [64], phenobarbital [65,66], lamotrigine [66,67], rufinamide [68], clobazam [69], clonazepam [69], levetiracetam [66], and carbamazepine [66,70]. DBS-based methods have also been applied in the TDM of anticancer drugs. Recently, Poetto et al. (2021) [71] developed and validated a dried blood spot LC-MS/MS method for the TDM of palbociclib, ribociclib, and letrozole in patients affected by cancer, and they observed a positive correlation between DBS and plasma concentrations for the three drugs. Berm et al. (2015) [48] presented a method for therapeutic drug monitoring of the tricyclic antidepressants amitriptyline, nortriptyline, imipramine, clomipramine, and their active metabolites in DBS using LC-MS/MS. The authors observed that a low hematocrit (≤30%) was associated with a negative bias (≥15%) for all analytes. In contrast, punching the blood spot sample from the perimeter instead of the center was associated with a positive bias. A good correlation was found between the patients’ plasma and DBS samples for all analytes except clomipramine.

DBS devices have been widely used in the TDM of immunosuppressants. Veenhof et al. (2023) [72] conducted a pilot proficiency test for the microsampling of immunosuppressants (tacrolimus, cyclosporine, everolimus, sirolimus, mycophenolic acid) involving 14 laboratories from seven countries in three rounds of proficiency testing. Immunosuppressant microsampling methods showed high interlaboratory variation compared with the whole blood methods, underscoring the need for harmonization and standardization. Proficiency testing should be routinely performed for laboratories using immunosuppressant microsampling techniques in patient care. In fact, Veenhof et al. (2019) [73] applied a DBS assay to measure sirolimus and everolimus in transplant patients. Passing–Bablok regression showed no significant differences between whole blood and DBS, but the limits or clinical significance were not reached (77.3% and 61.5%, respectively). In an effort to reduce the hematocrit effect and volume problem that plague DBS, devices capable of collecting definite volume samples have been designed [74], such as disposable low-cost viable capillaries [75] and DBS with metering capillary channels. Alternatives for an Hct-independent determination of drugs in blood microsamples are the Volumetric Absorptive Paper Minidiscs (VAPD-mini) [76] and the Hemapen^®^ [77].

A popular technique to control the volume of blood microsamples is volumetric absorptive microsampling (VAMS), a device consisting of a globular hydrophilic tip mounted on a plastic tip to collect a fixed volume of sample [78,79,80]. In 2014, Neoteryx commercialized a microdevice called Mitra^®^, based on the principle of VAMS [80], which has been designed to present all the advantages of the DBS technique without the effect of hematocrit, simplifying the workflow for the analysis of whole blood samples [81]. Different configurations of VAMS device are available, allowing 10, 20, and 30 µL of whole blood to be collected [80]. A finger or heel prick is made, then the adsorbent sampling tip is placed in contact with only the surface of the head of the tip in the blood drop [80,82]. The tip is inserted into the blood drop, allowing adsorption by capillarity. The tip must be held in contact with the blood drop for approximately 2–3 s to allow complete filling [82]. Contact times longer than 6 s may alter the volume collected by overfilling the tip. The VAMS method results were accurate and reproducible, even under home sampling conditions [83]. From the standpoint of home sampling, where collection is carried out without the help of trained health care personnel, adequate training on how to sample with VAMS is extremely critical to ensure a good sample quality. An often-adopted solution, which has proven successful, is to provide training video tutorials and instructions online [80,82,84]. Several studies have demonstrated the low impact of Hct on the analytical performance of VAMS [85,86,87,88,89,90].

Again, a comparison of plasma/blood and VAMS methods is needed to apply them in the clinical setting. For cannabidiol (CBD) and its main metabolites, it has been shown that concentrations in VAMS devices and plasma are not significantly different [91,92]. Therapeutic drug monitoring of blood levels of cannabinoids is crucial for optimizing the medical cannabis therapy, and the use of microsampling devices could facilitate the widespread adoption of this clinical practice, as well as simplify the sampling in patients who are not compliant with venipuncture. Another technique for collecting blood microsamples is three-dimensional (3D) dried blood spheroids (3D-DBS), a device based on hydrophobic papers, in contrast to traditional planar (2D) hydrophilic cellulose-based papers. Cellulose is functionalized with trichloro(3,3,3-trifluoropropyl)silane. Aqueous blood samples are deposited on the surface as droplets, leading to the formation of 3D-DBS. The blood spheroids form a barrier between the analytes and air that protects the analytes from oxidative degradation and thermal conduction [93]. Paper functionalization has recently been exploited also for the production of molecularly imprinted-interpenetrating polymer network (MI-IPN) devices [94].

## 3. Plasma Microsamples

Plasma and/or whole blood represent the “gold standard” matrices for TDM [2]. The use of plasma or blood as a matrix depends on the distribution characteristics of the drugs. DPS is obtained by spotting the plasma obtained after laboratory centrifugation of very small quantities of blood on classic cellulose paper substrates or on special glass substrates [95]. DPS presents all logistic and managerial advantages of DBS [8] but is not affected by the Hct effect [96]. In this case, the disadvantage is the need to perform a conventional venipuncture, which, therefore, requires qualified healthcare personnel and a laboratory that processes the whole blood sample by centrifuging it, separating the plasma, and identifying a known volume of blood on the card. For these reasons, the DPS is useful when there is a need to send the sample to an external laboratory, for example, in multi-center studies or in non-standard hospitals, because dry samples are usually more stable than fresh ones, but it does not avoid the inconvenience caused by venipuncture and the need for patients to move to a hospital for blood collection [97]. Recently, devices have been introduced on the market that allow the collection of DPS without the need for centrifugation. In these devices, whole blood is filtered by the action of capillary forces through passive microfluidics, and the excess sample is drained to avoid overfilling, thus allowing a known volume of blood to be collected [98]. Other self-contained microfluidic plasma sampling devices consist of two layers of material: an asymmetric polymer membrane that serves as a filter for red blood cells and a cellulose layer responsible for absorbing plasma from the first layer [99]. TDM based on DPS is a clinical practice for some classes of drugs, such as anti-epileptics [7,100], antibiotics [8,101,102], antivirals [5], antipsychotics [63], antiretrovirals [103,104], and amantadine hydrochloride [105].

In our opinion, these innovative DPS devices offer an attractive alternative to traditional plasma samples for TDM, and the new devices with filter membranes could be useful for the development of home sampling strategies.

## 4. Urine Microsamples

Urine is an excreted biological fluid and requires simple, noninvasive collection because it does not require skin puncture. Plasma and/or whole blood are the gold standard matrices for TDM, while urine is widely used in forensic toxicology for the identification of illicit substances [106]. Urine is not a sample of choice for TDM, but urinary TDM has applications in some specific contexts, for example, to verify treatment compliance and therapeutic adherence and to identify cases of abuse [106,107,108,109]. TDM in urinary samples can also be used to study the urinary disposition of drugs with renal toxicity, where drug penetration might be a predictor of renal damage [110].

Dried urine microsamples (DUS) are easily obtained by spotting a drop of the urine sample onto a filter paper and then drying it. Although stability must be evaluated analyte by analyte, as with other microsampling methods, DUSs, being dry, usually offer greater analyte stability than fresh urine, reducing shipping costs. They have been tested in different applications, such as newborn screening [111], clinical diagnosis of diseases such as metabolic disorders [112], and clinical assessments of urinary hormone disorders [113], and they can be very useful in the determination of illicit drug substances and their metabolites [114]. In pharmacology, urine is a helpful alternative matrix in special cases, such as in elderly patients who are taking multiple medications since the presence of metabolites in the urine depends on metabolic pathways and the patients’ renal function [115,116]. They have been applied to antimicrobials [117], antiparkinsonians [118], antivirals, and antiretrovirals, in particular to predict tenofovir nephrotoxicity and to tailor the appropriate dosage of this drug [110]. VAMS spotted with urine have been studied [80] in the field of the anti-doping analysis of glucocorticoids, such as cortisol in urine samples from athletes. Three matrices were compared: urine, VAMS spotted with urine, and DUS. For VAMS spotted with urine, a higher extraction accuracy and yield were found; however, the study should be repeated by analyzing a greater sample number [80,119].

## 5. Breast Milk Microsamples

Breast milk is not a conventional sampling matrix for TDM, but the study of drug penetration in breast milk is a key aspect in directing breastfeeding mothers toward the best drug therapy. The availability of analytical methods that allow the assay of drugs in human breast milk can be very useful for ensuring appropriate dosage of maternal drugs and reducing the risk of adverse drug reactions in breastfed babies [120,121,122,123]. The possible risk of drug-induced toxicity in breastfed infants can be predicted based on the milk–plasma ratio [2]. Antiretrovirals [124], antidepressants [125], antihypertensives [126], antipsychotics, opioids, benzodiazepines, nicotine, caffeine, and alcohol [122] have been determined in breastfeeding women to evaluate the risk for infants to be exposed to these therapeutic agents through lactation. Sample collection is noninvasive and simple, but breast milk is a complex matrix rich in proteins, carbohydrates, and fats, which requires a relatively complete extraction process to achieve higher recovery analytical results [2]. From the analytical point of view, dried milk breast spots (DBMS) can help overcome drug extraction issues. DMBS present the same logistic advantages of other dried spot microsamples. DMBS have been used for studying the complete PK profiles of efavirenz in human breast milk and for the TDM of other antiretrovirals [127], such as lamivudine, emtricitabine, tenofovir [17,128], and nevirapine [129]. It has also been used for the quantification of the rheumatoid arthritis therapy agent tocilizumab [130] and antidepressants [131]. Muller et al. (2013) [132] studied the concentration of sertraline in breast milk and breastfed infants by LC-MS/MS analysis. O’Halloran et al. [123] quantified amisulpride in breast milk by LC-MS/MS and found high concentrations of the drug in breast milk, resulting in therapeutic levels of this drug in the infant with potential toxic effects.

## 6. Saliva Microsamples

Saliva contains only the free fraction of drugs, which can infiltrate through salivary tissues [133,134], so the concentration of drugs in saliva is strongly correlated with the therapeutically active fraction of the drug [134]. Saliva, or oral fluid, is an emerging matrix among biological fluids because it has many advantages over traditional venipuncture. Therefore, saliva has been increasingly used for the therapeutic monitoring of several drugs [133,135], such as codeine phosphate [136], carbamazepine [137,138,139], phenytoin, phenobarbital [139,140], and primidone [139,140], demonstrating a relationship between drug concentrations in saliva and plasma [141]. Saliva outperforms traditional plasma/blood sampling in terms of ease of use for the patient, allowing noninvasive, safe, and painless sampling that is easily applicable to home self-sampling [141,142]. However, in saliva, debris and contamination from food intake can affect the concentration of the analytes, and the nonsterility increases the risk of bacterial degradation of the analytes during long-term storage, especially in the absence of refrigeration during transport [141]. Dried saliva samples (DSS) can be a solution to overcome this issue. DSS are obtained by staining collected saliva on pure cellulose filter paper. The saliva collection paper is allowed to dry and is then stored at room temperature. In fact, the saliva and target analytes adhere to the filter paper, increasing the stability of the saliva sample [141]. It has been demonstrated that alginate- and chitosan-treated papers further improved the sample stability for up to 30 days [143]. This approach has proven useful in the assay of the oral cancer biomarker (matrix metalloproteinase-1) [144] in the diagnosis of congenital cytomegalovirus [145] and for the measurement of antiepileptic [18,133], cannabinoids [146], and metabolites [103,147,148].

In addition, VAMS devices have been applied to saliva samples [80]. Marasca et al. (2020) [149] used saliva to quantify antidepressants, but VAMS concentrations in saliva were significantly higher than VAMS concentrations in whole blood, and they found no correlation between blood and saliva levels.

## 7. Conclusions

The main limitation of microsampling is the high cost of instrumentation capable of quantifying analytes in very small volumes with sufficient sensitivity, such as liquid chromatography coupled with tandem mass spectrometry (LC-MS/MS), which is found only in specialized centers. In addition, it is necessary to validate the specific reference/target intervals for each microsampling device. However, the use of microsampling usually has the advantage of higher stability of the analytes, to be evaluated on a case-by-case basis, and, thus, facilitates shipping because of both lower costs and a lower risk of sample damage. Microsampling can, thus, ensure more equal access to TDM and an optimization of the therapies. Moreover, the availability of new microsampling devices that allow home sampling collection—avoiding venipuncture and collecting a known volume of sample and eliminating the need for patients to move to a hospital for blood collection—could lead to a reduction in public health system costs and prove to be time-saving for patients. Table 1 shows a summary of the microsampling techniques described with the relevant literature references.

## Figures and Tables

**Table 1:** Summary of microsampling devices in different matrices and relative references.

Matrix	Microsampling Devices	References
Blood	Dried Blood Spot	[11,38,39,40,41,42,43,44,45,46,47,48,49,50,51,52,53,54,55,56,57,58,59,60,61,62,64,65,66,67,68,69,70,71,72,73,74,75]
	Volumetric Absorptive Paper Minidiscs (VAPD-mini)	[76]
	Hemapen^®^	[77]
	VAMS	[78,79,80,85,86,87,88,89,90,91,92,93,94,96]
Plasma	Dried Plasma Spot	[5,7,8,63,71,95,96,97,98,99,100,101,102,103,104,105,117]
	Novel membrane devices	[98]
Urine	Dried Urine Spot	[108,109,110,111,112,113,114,115,116,117,118]
	VAMS spotted with urine	[80,119]
Breast Milk	Dried Breast Milk Spot	[16,120,121,122,124,125,126,127,128,129,130,131,132]
Saliva	Dried Saliva Spot	[18,135,137,138,140,141,143,144,145,146,147,148]
	VAMS spotted with saliva	[149]

## Data Availability

Not applicable.
